# 4-Methyl-*N*-[(*Z*)-3-(4-methyl­phen­ylsulfon­yl)-1,3-thia­zolidin-2-yl­idene]benzene­sulfonamide

**DOI:** 10.1107/S1600536811028807

**Published:** 2011-07-23

**Authors:** Hui-Ling Hu, Geng-Ren Yang, Chun-Wei Yeh

**Affiliations:** a Department of Chemical Engineering and Material Engineering, Graduate School of Materials Applied Technology, Nanya Institute of Technology, Chung-Li, Taiwan; b Department of Chemistry, Chung-Yuan Christian University, Chung-Li, Taiwan

## Abstract

In the crystal structure of the title compound, C_17_H_18_N_2_O_4_S_3_, mol­ecules are connected into centrosymmetric dimers *via* weak inter­molecular C—H⋯π inter­actions. These dimers are further connected through a series of weak C—H⋯O hydrogen bonds, while futher C—H⋯π inter­actions involving the phenyl and thia­zoline rings are also observed. The thia­zolidine ring is twisted from the benzene rings rings by dihedral angles of 79.1 (1) and 85.0 (1)°, while the dihedral angle between two benzene rings is 76.0 (1)°.

## Related literature

For background to *N*-heterocyclic sulfanilamide derivatives, see: Kuz’mina *et al.* (1962 ▶); Jensen & Thorsteinsson (1941 ▶); Hunter & Kolloff (1943 ▶); Hultquist *et al.* (1951 ▶). For a related synthesis, see: Razvodovskaya *et al.* (1990 ▶). 
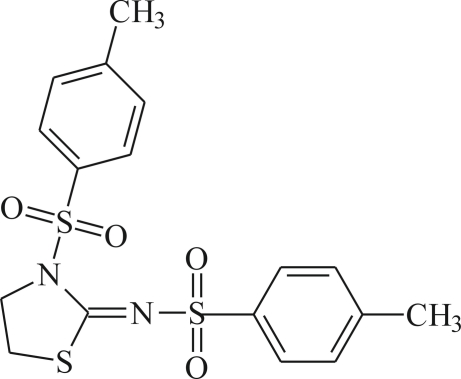


## Experimental

### 

#### Crystal data

C_17_H_18_N_2_O_4_S_3_
*M* *_r_* = 410.51
Monoclinic, 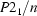

*a* = 9.3825 (2) Å
*b* = 14.4047 (2) Å
*c* = 14.2279 (3) Å
β = 102.666 (1)°
*V* = 1876.14 (6) Å^3^
*Z* = 4
Mo *K*α radiation
μ = 0.42 mm^−1^
*T* = 296 K
0.40 × 0.40 × 0.40 mm

#### Data collection

Bruker APEXII CCD diffractometer
Absorption correction: multi-scan (*SADABS*; Bruker, 2001 ▶) *T*_min_ = 0.845, *T*_max_ = 0.845
17749 measured reflections
4652 independent reflections
3756 reflections with *I* > 2σ(*I*)
*R*_int_ = 0.025

#### Refinement

*R*[*F* ^2^ > 2σ(*F* ^2^)] = 0.037
*wR*(*F* ^2^) = 0.107
*S* = 1.04
4652 reflections
236 parameters
H-atom parameters constrained
Δρ_max_ = 0.29 e Å^−3^
Δρ_min_ = −0.29 e Å^−3^

### 

Data collection: *APEX2* (Bruker, 2010 ▶); cell refinement: *SAINT* (Bruker, 2010 ▶); data reduction: *SAINT*; program(s) used to solve structure: *SHELXS97* (Sheldrick, 2008 ▶); program(s) used to refine structure: *SHELXL97* (Sheldrick, 2008 ▶); molecular graphics: *SHELXTL* (Sheldrick, 2008 ▶); software used to prepare material for publication: *SHELXL97*.

## Supplementary Material

Crystal structure: contains datablock(s) I, global. DOI: 10.1107/S1600536811028807/fj2445sup1.cif

Structure factors: contains datablock(s) I. DOI: 10.1107/S1600536811028807/fj2445Isup2.hkl

Supplementary material file. DOI: 10.1107/S1600536811028807/fj2445Isup3.cml

Additional supplementary materials:  crystallographic information; 3D view; checkCIF report

## Figures and Tables

**Table 1:** Hydrogen-bond geometry (Å, °) *Cg*1 and *Cg*2 are the centroids of the C4–C9 and C11–C16 benzene rings, respectively.

*D*—H⋯*A*	*D*—H	H⋯*A*	*D*⋯*A*	*D*—H⋯*A*
C10—H10*B*⋯*Cg*1^i^	0.97	2.91	3.567 (1)	127
C2—H2*B*⋯*Cg*2^ii^	0.97	3.09	3.821 (1)	134
C1—H1*B*⋯O1^ii^	0.97	2.59	3.394 (3)	141
C12—H12*A*⋯O3^iii^	0.93	2.47	3.318 (2)	151

Symmetry codes: (i) 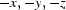; (ii) 
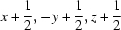
; (iii) 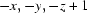.

**Fig. 1.**
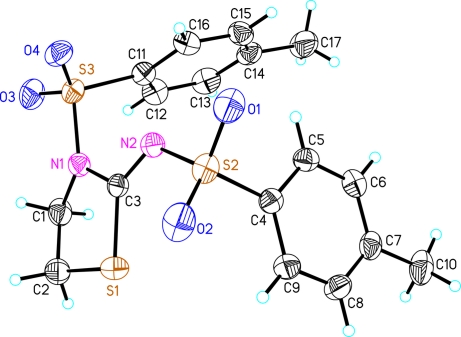
Crystal structure of the title compound with atom labeling and displacement ellipsoids drawn at the 30% probability level.

## supplementary crystallographic information

### Comment

In a series of N-heterocyclic sulfanilamide derivatives which prepared and are investigating biologically one of the compounds, 2-sulfanilyl-aminothiazoline, proved to be of particular interest, both chemically and therapeutically. (Kuz'mina *et al.*, 1962; Jensen *et al.*, 1941; Hunter *et al.*, 1943; Hultquist *et al.*, 1951). The synthesis and character the 3-substituted 2-(thiophosphorylimino)thiazolidine compounds are also reported (Razvodovskaya *et al.*, 1990). Within this project the crystal structure of the title compound was determined. The crystal structure features inversion-related dimers linked by the weak intermolecular C—H···pi interactions in the solid state, while *Cg1* and *Cg2* are the centers of C4—C9 and C11—C16 and these carbon atoms of mean devition from plane are 0.0008 and 0.0043 Å. Weak C—H···O hydrogen bonds among the molecules are also observed in the solid state. The thiazolidine and the phenyl rings are not coplanar but twisted with each other by an interplanar angles of 79.1 (1) and 85.0 (1)°, respectively, while the dihedral angle between two phenyl groups is 76.0 (1)°.

### Experimental

The title compound was prepared according to a published procedure (Razvodovskaya *et al.*, 1990). Block like crystals suitable for X-ray crystallography were obtained by slow evaporization of the solvent from a solution of the title compound in methanol.

### Refinement

All the hydrogen atoms were discernible in the difference Fourier maps. However, they were situated into the idealized positions and constrained by the riding atom approximation: *C*—Hmethyl = 0.96 Å and *C*—Hmethylene = 0.97 Å while the methyls and methylenes were allowed to rotate about their respective axes; *C*—Haryl = 0.93 Å; *U*_iso_(Hmethyl) = 1.5*U*_eq_(Cmethyl); *U*_iso_(Haryl or methylene) = 1.2*U*_eq_(Caryl or methylene).

### Crystal data

**Table:**

C_17_H_18_N_2_O_4_S_3_	*F*(000) = 856
*M**_r_* = 410.51	*D*_x_ = 1.453 Mg m^−^^3^
Monoclinic, *P*2_1_/*n*	Mo *K*α radiation, λ = 0.71073 Å
Hall symbol: -P 2yn	Cell parameters from 8638 reflections
*a* = 9.3825 (2) Å	θ = 2.4–28.2°
*b* = 14.4047 (2) Å	µ = 0.42 mm^−^^1^
*c* = 14.2279 (3) Å	*T* = 296 K
β = 102.666 (1)°	Block, colourless
*V* = 1876.14 (6) Å^3^	0.40 × 0.40 × 0.40 mm
*Z* = 4	

### Data collection

**Table:**

Bruker APEXII CCD diffractometer	4652 independent reflections
Radiation source: fine-focus sealed tube	3756 reflections with *I* > 2σ(*I*)
graphite	*R*_int_ = 0.025
phi and ω scans	θ_max_ = 28.3°, θ_min_ = 2.0°
Absorption correction: multi-scan (*SADABS*; Bruker, 2001)	*h* = −12→12
*T*_min_ = 0.845, *T*_max_ = 0.845	*k* = −15→19
17749 measured reflections	*l* = −17→18

### Refinement

**Table:**

Refinement on *F*^2^	Secondary atom site location: difference Fourier map
Least-squares matrix: full	Hydrogen site location: inferred from neighbouring sites
*R*[*F*^2^ > 2σ(*F*^2^)] = 0.037	H-atom parameters constrained
*wR*(*F*^2^) = 0.107	*w* = 1/[σ^2^(*F*_o_^2^) + (0.0497*P*)^2^ + 0.6237*P*] where *P* = (*F*_o_^2^ + 2*F*_c_^2^)/3
*S* = 1.04	(Δ/σ)_max_ < 0.001
4652 reflections	Δρ_max_ = 0.29 e Å^−^^3^
236 parameters	Δρ_min_ = −0.29 e Å^−^^3^
0 restraints	Extinction correction: *SHELXL*, Fc^*^=kFc[1+0.001xFc^2^λ^3^/sin(2θ)]^-1/4^
Primary atom site location: structure-invariant direct methods	Extinction coefficient: 0.0091 (9)

### Special details

**Table:**

Geometry. All e.s.d.'s (except the e.s.d. in the dihedral angle between two l.s. planes) are estimated using the full covariance matrix. The cell e.s.d.'s are taken into account individually in the estimation of e.s.d.'s in distances, angles and torsion angles; correlations between e.s.d.'s in cell parameters are only used when they are defined by crystal symmetry. An approximate (isotropic) treatment of cell e.s.d.'s is used for estimating e.s.d.'s involving l.s. planes.
Refinement. Refinement of *F*^2^ against ALL reflections. The weighted *R*-factor *wR* and goodness of fit *S* are based on *F*^2^, conventional *R*-factors *R* are based on *F*, with *F* set to zero for negative *F*^2^. The threshold expression of *F*^2^ > σ(*F*^2^) is used only for calculating *R*-factors(gt) *etc*. and is not relevant to the choice of reflections for refinement. *R*-factors based on *F*^2^ are statistically about twice as large as those based on *F*, and *R*- factors based on ALL data will be even larger.

### Fractional atomic coordinates and isotropic or equivalent isotropic displacement parameters (Å^2^)

**Table:**

	*x*	*y*	*z*	*U*_iso_*/*U*_eq_	
N1	0.16502 (15)	0.17067 (10)	0.38640 (10)	0.0446 (3)	
N2	0.08736 (15)	0.26664 (10)	0.25505 (10)	0.0448 (3)	
S1	0.34585 (5)	0.17353 (4)	0.27357 (4)	0.06158 (16)	
S2	0.10549 (5)	0.31474 (3)	0.15432 (3)	0.04983 (13)	
S3	0.01808 (5)	0.19186 (3)	0.43270 (3)	0.04805 (13)	
O1	−0.00727 (19)	0.38361 (9)	0.13422 (10)	0.0693 (4)	
O2	0.25206 (18)	0.34381 (12)	0.15775 (11)	0.0739 (4)	
O3	0.04496 (18)	0.13807 (11)	0.51854 (10)	0.0702 (4)	
O4	0.00217 (15)	0.28956 (9)	0.43817 (10)	0.0580 (3)	
C1	0.2585 (2)	0.08847 (14)	0.41762 (15)	0.0590 (5)	
H1A	0.2091	0.0321	0.3909	0.071*	
H1B	0.2818	0.0836	0.4873	0.071*	
C2	0.3947 (2)	0.10280 (16)	0.38077 (16)	0.0664 (6)	
H2A	0.4335	0.0436	0.3656	0.080*	
H2B	0.4684	0.1338	0.4290	0.080*	
C3	0.18420 (17)	0.21042 (11)	0.30207 (11)	0.0407 (3)	
C4	0.06041 (19)	0.22754 (11)	0.06650 (12)	0.0431 (4)	
C5	−0.08309 (19)	0.19554 (12)	0.04084 (13)	0.0481 (4)	
H5A	−0.1533	0.2177	0.0723	0.058*	
C6	−0.1198 (2)	0.13072 (13)	−0.03153 (14)	0.0534 (4)	
H6A	−0.2156	0.1094	−0.0486	0.064*	
C7	−0.0173 (2)	0.09647 (13)	−0.07954 (13)	0.0542 (4)	
C8	0.1244 (2)	0.12907 (15)	−0.05287 (14)	0.0607 (5)	
H8A	0.1943	0.1068	−0.0844	0.073*	
C9	0.1647 (2)	0.19422 (14)	0.01986 (14)	0.0538 (4)	
H9A	0.2606	0.2152	0.0370	0.065*	
C10	−0.0606 (3)	0.02670 (16)	−0.15964 (16)	0.0787 (7)	
H10A	−0.1623	0.0119	−0.1675	0.118*	
H10B	−0.0034	−0.0287	−0.1439	0.118*	
H10C	−0.0438	0.0524	−0.2185	0.118*	
C11	−0.12963 (18)	0.14496 (12)	0.34887 (12)	0.0460 (4)	
C12	−0.1544 (2)	0.04982 (13)	0.35063 (15)	0.0578 (5)	
H12A	−0.0965	0.0124	0.3971	0.069*	
C13	−0.2660 (2)	0.01228 (15)	0.28240 (17)	0.0652 (5)	
H13A	−0.2837	−0.0512	0.2836	0.078*	
C14	−0.3526 (2)	0.06605 (15)	0.21206 (15)	0.0579 (5)	
C15	−0.3267 (2)	0.16119 (15)	0.21243 (15)	0.0557 (5)	
H15A	−0.3850	0.1986	0.1661	0.067*	
C16	−0.21635 (19)	0.20075 (13)	0.28026 (14)	0.0507 (4)	
H16A	−0.2002	0.2644	0.2800	0.061*	
C17	−0.4719 (3)	0.0231 (2)	0.13607 (19)	0.0864 (8)	
H17A	−0.4738	−0.0427	0.1461	0.130*	
H17B	−0.4536	0.0354	0.0735	0.130*	
H17C	−0.5644	0.0494	0.1403	0.130*	

### Atomic displacement parameters (Å^2^)

**Table:**

	*U*^11^	*U*^22^	*U*^33^	*U*^12^	*U*^13^	*U*^23^
N1	0.0461 (7)	0.0435 (7)	0.0418 (7)	0.0049 (6)	0.0045 (6)	−0.0002 (6)
N2	0.0453 (7)	0.0459 (8)	0.0429 (7)	0.0017 (6)	0.0095 (6)	0.0013 (6)
S1	0.0508 (3)	0.0734 (3)	0.0623 (3)	0.0140 (2)	0.0163 (2)	−0.0073 (2)
S2	0.0627 (3)	0.0401 (2)	0.0470 (2)	−0.00536 (18)	0.0128 (2)	0.00129 (17)
S3	0.0555 (3)	0.0491 (3)	0.0406 (2)	−0.00057 (18)	0.01281 (18)	−0.00071 (17)
O1	0.1039 (12)	0.0425 (7)	0.0589 (8)	0.0189 (7)	0.0122 (8)	0.0043 (6)
O2	0.0802 (10)	0.0758 (10)	0.0684 (9)	−0.0367 (8)	0.0217 (8)	−0.0044 (8)
O3	0.0878 (11)	0.0805 (10)	0.0427 (7)	0.0010 (8)	0.0149 (7)	0.0108 (7)
O4	0.0645 (8)	0.0504 (7)	0.0619 (8)	0.0006 (6)	0.0197 (7)	−0.0130 (6)
C1	0.0684 (12)	0.0496 (10)	0.0521 (10)	0.0146 (9)	−0.0017 (9)	0.0011 (8)
C2	0.0637 (12)	0.0655 (13)	0.0631 (12)	0.0251 (10)	−0.0009 (10)	−0.0143 (10)
C3	0.0399 (8)	0.0402 (8)	0.0402 (8)	−0.0027 (6)	0.0052 (6)	−0.0082 (6)
C4	0.0484 (9)	0.0396 (8)	0.0416 (8)	0.0018 (7)	0.0110 (7)	0.0055 (7)
C5	0.0472 (9)	0.0468 (9)	0.0520 (10)	0.0050 (7)	0.0144 (7)	0.0031 (8)
C6	0.0529 (10)	0.0479 (10)	0.0546 (10)	−0.0025 (8)	0.0014 (8)	0.0027 (8)
C7	0.0726 (12)	0.0458 (10)	0.0403 (9)	0.0106 (9)	0.0041 (8)	0.0038 (7)
C8	0.0659 (12)	0.0680 (13)	0.0519 (11)	0.0153 (10)	0.0210 (9)	−0.0008 (9)
C9	0.0484 (9)	0.0636 (11)	0.0512 (10)	0.0007 (8)	0.0153 (8)	0.0020 (8)
C10	0.1124 (19)	0.0631 (13)	0.0513 (12)	0.0143 (13)	−0.0025 (12)	−0.0083 (10)
C11	0.0473 (9)	0.0452 (9)	0.0478 (9)	−0.0052 (7)	0.0155 (7)	0.0038 (7)
C12	0.0651 (12)	0.0470 (10)	0.0626 (12)	−0.0066 (9)	0.0171 (9)	0.0100 (9)
C13	0.0710 (13)	0.0470 (11)	0.0826 (15)	−0.0152 (9)	0.0279 (11)	−0.0052 (10)
C14	0.0491 (10)	0.0687 (12)	0.0607 (11)	−0.0102 (9)	0.0226 (9)	−0.0147 (10)
C15	0.0441 (9)	0.0665 (12)	0.0578 (11)	−0.0001 (8)	0.0139 (8)	0.0053 (9)
C16	0.0467 (9)	0.0451 (9)	0.0622 (11)	−0.0022 (7)	0.0157 (8)	0.0064 (8)
C17	0.0689 (14)	0.0996 (19)	0.0894 (18)	−0.0144 (13)	0.0148 (13)	−0.0413 (15)

### Geometric parameters (Å, °)

**Table:**

N1—C3	1.376 (2)	C6—H6A	0.9300
N1—C1	1.483 (2)	C7—C8	1.382 (3)
N1—S3	1.6811 (15)	C7—C10	1.507 (3)
N2—C3	1.289 (2)	C8—C9	1.387 (3)
N2—S2	1.6340 (15)	C8—H8A	0.9300
S1—C3	1.7368 (16)	C9—H9A	0.9300
S1—C2	1.808 (2)	C10—H10A	0.9600
S2—O2	1.4282 (15)	C10—H10B	0.9600
S2—O1	1.4327 (15)	C10—H10C	0.9600
S2—C4	1.7566 (17)	C11—C16	1.383 (3)
S3—O4	1.4192 (14)	C11—C12	1.391 (3)
S3—O3	1.4215 (14)	C12—C13	1.373 (3)
S3—C11	1.7536 (18)	C12—H12A	0.9300
C1—C2	1.498 (3)	C13—C14	1.380 (3)
C1—H1A	0.9700	C13—H13A	0.9300
C1—H1B	0.9700	C14—C15	1.392 (3)
C2—H2A	0.9700	C14—C17	1.508 (3)
C2—H2B	0.9700	C15—C16	1.375 (3)
C4—C9	1.383 (2)	C15—H15A	0.9300
C4—C5	1.394 (2)	C16—H16A	0.9300
C5—C6	1.377 (3)	C17—H17A	0.9600
C5—H5A	0.9300	C17—H17B	0.9600
C6—C7	1.386 (3)	C17—H17C	0.9600
			
C3—N1—C1	114.30 (15)	C7—C6—H6A	119.2
C3—N1—S3	122.77 (11)	C8—C7—C6	118.23 (17)
C1—N1—S3	120.66 (13)	C8—C7—C10	121.2 (2)
C3—N2—S2	121.62 (12)	C6—C7—C10	120.6 (2)
C3—S1—C2	92.76 (9)	C7—C8—C9	121.55 (18)
O2—S2—O1	117.85 (10)	C7—C8—H8A	119.2
O2—S2—N2	112.27 (9)	C9—C8—H8A	119.2
O1—S2—N2	104.73 (8)	C4—C9—C8	119.15 (18)
O2—S2—C4	108.29 (9)	C4—C9—H9A	120.4
O1—S2—C4	107.52 (9)	C8—C9—H9A	120.4
N2—S2—C4	105.40 (8)	C7—C10—H10A	109.5
O4—S3—O3	119.63 (9)	C7—C10—H10B	109.5
O4—S3—N1	107.86 (8)	H10A—C10—H10B	109.5
O3—S3—N1	103.39 (8)	C7—C10—H10C	109.5
O4—S3—C11	110.02 (9)	H10A—C10—H10C	109.5
O3—S3—C11	109.83 (9)	H10B—C10—H10C	109.5
N1—S3—C11	104.90 (8)	C16—C11—C12	120.69 (18)
N1—C1—C2	106.21 (17)	C16—C11—S3	120.74 (14)
N1—C1—H1A	110.5	C12—C11—S3	118.52 (15)
C2—C1—H1A	110.5	C13—C12—C11	118.67 (19)
N1—C1—H1B	110.5	C13—C12—H12A	120.7
C2—C1—H1B	110.5	C11—C12—H12A	120.7
H1A—C1—H1B	108.7	C12—C13—C14	121.87 (19)
C1—C2—S1	107.17 (13)	C12—C13—H13A	119.1
C1—C2—H2A	110.3	C14—C13—H13A	119.1
S1—C2—H2A	110.3	C13—C14—C15	118.41 (19)
C1—C2—H2B	110.3	C13—C14—C17	121.1 (2)
S1—C2—H2B	110.3	C15—C14—C17	120.5 (2)
H2A—C2—H2B	108.5	C16—C15—C14	120.96 (19)
N2—C3—N1	120.10 (15)	C16—C15—H15A	119.5
N2—C3—S1	128.55 (13)	C14—C15—H15A	119.5
N1—C3—S1	111.35 (12)	C15—C16—C11	119.39 (17)
C9—C4—C5	120.25 (17)	C15—C16—H16A	120.3
C9—C4—S2	120.28 (14)	C11—C16—H16A	120.3
C5—C4—S2	119.40 (13)	C14—C17—H17A	109.5
C6—C5—C4	119.28 (17)	C14—C17—H17B	109.5
C6—C5—H5A	120.4	H17A—C17—H17B	109.5
C4—C5—H5A	120.4	C14—C17—H17C	109.5
C5—C6—C7	121.53 (18)	H17A—C17—H17C	109.5
C5—C6—H6A	119.2	H17B—C17—H17C	109.5

### Hydrogen-bond geometry (Å, °)

**Table:**

Cg1 and Cg2 are the centroids of the C4–C9 and C11–C16 benzene rings, respectively.

**Table:**

*D*—H···*A*	*D*—H	H···*A*	*D*···*A*	*D*—H···*A*
C10—H10B···Cg1^i^	0.97	2.91	3.567 (1)	127
C2—H2B···Cg2^ii^	0.97	3.09	3.821 (1)	134
C1—H1B···O1^ii^	0.97	2.59	3.394 (3)	141
C12—H12A···O3^iii^	0.93	2.47	3.318 (2)	151

Symmetry codes: (i) −*x*, −*y*, −*z*; (ii) *x*+1/2, −*y*+1/2, *z*+1/2; (iii) −*x*, −*y*, −*z*+1.

## References

[bb1] Bruker (2001). *SADABS.* Bruker AXS Inc., Madison, Wisconsin, USA.

[bb2] Bruker (2010). *APEX2* and *SAINT* Bruker AXS Inc., Madison, Wisconsin, USA.

[bb3] Hultquist, M. E., Germann, R. P., Webb, J. S., Wright, W. B. Jr, Roth, B., Smith, J. M. Jr & SubbaRow, Y. (1951). *J. Am. Chem. Soc.* **73**, 2558–2566.

[bb4] Hunter, J. H. & Kolloff, H. G. (1943). *J. Am. Chem. Soc.* **65**, 156–159.

[bb5] Jensen, K. A. & Thorsteinsson, T. (1941). *Dansk Tidsskrift* *Farmaci*, **15**, 41–77.

[bb6] Kuz’mina, K. K., Ostroumova, N. G., Markova, Yu. V. & Shchukina, M. N. (1962). *Zhurnal Obshchei Khimii.* **32**, 3390–3393.

[bb7] Razvodovskaya, L. V., Vorob’eva, N. N., Grapov, A. F. & Mel’nikov, N. N. (1990). *Zhurnal Obshchei Khimii*, **60**, 1518–1525.

[bb8] Sheldrick, G. M. (2008). *Acta Cryst.* A**64**, 112–122. 10.1107/S0108767307043930 18156677

